# Open-heart surgery and coronary artery bypass grafting in Western Africa

**DOI:** 10.4314/pamj.v9i1.71175

**Published:** 2011-05-11

**Authors:** Frank Edwin, Kwabena Frimpong-Boateng

**Affiliations:** 1 National Cardiothoracic Center, Korle Bu Teaching Hospital, PO Box KB 846, Accra, Ghana; 2 Walter Sisulu Pediatric Cardiac Center, Sunninghill Hospital, Johannesburg, South Africa

**Keywords:** Open-heart surgery, cardiothoracic surgery, Africa

## Abstract

We read with concern the paper of Budzee and colleagues in a recent issue of the Pan African Medical Journal. We wish to draw the attention of the authors and the readership of the journal to gross inaccuracies in the report. The first open-heart surgery in Nigeria is reported to have taken place on 1^st^ February 1974 at the University of Nigeria Teaching Hospital (UNTH) in Enugu. Publications from the group in Abidjan indicate the performance of the first 300 cases of open-heart surgery by 1983, the figure increasing to 850 by 1987. Senegal reportedly began performing open-heart surgery in 1995 and is currently a reference point for open cardiac procedures for francophone West Africa. The Ghanaian open-heart experience began in 1964 when surface cooling was used to achieve hypothermia for the successful closure of an atrial septal defect. However, it was not until 1989 that Ghana's National Cardiothoracic Center (NCTC) was established. The NCTC performs regular open-cardiac procedures covering almost the entire spectrum of cardiothoracic procedures including video-assisted thoracoscopic surgery (VATS). The NCTC is equipped with modern cardiovascular/thoracic facilities and has been accredited by the West African College of Surgeons as a center of excellence for the training of cardiothoracic surgeons and has performed creditably in this regard. It is emphasized that open-heart surgery has been practiced in West Africa for decades and continues to be practiced with excellence matching international standards at Ghana's National Cardiothoracic Center.

## To the editors of the Pan African Medical Journal

We read with concern the paper of Budzee and colleagues in a recent issue of the Pan African Medical Journal [1]. We wish to draw the attention of the authors and the readership of the journal to gross inaccuracies in the report [1].

The West African sub-region is deficient in terms of healthcare facilities, especially those relating to cardiac surgery. However, open heart surgery is not new in the sub-region neither is coronary artery bypass graft, as Budzee and colleagues claim in their publication [1]. It is disquieting when the authors claim that the Shisong Center in Cameroon is the only cardio-surgical center in Central / West Africa equipped with ultra modern health technologies offering diverse services [1,2]. This is particularly so when one considers that immediately west of Cameroon, the first open-heart surgery in neighboring Nigeria was reported to have taken place on 1st February 1974 at the University of Nigeria Teaching Hospital (UNTH) in Enugu [3]. By the year 2000, a total of 102 such operations had been carried out at the center by different Nigerian teams. At the time of Eze and Ezemba's report [3], three government-owned centers were practicing open heart surgery in Nigeria, the UNTH group being the most active. Also, as far back as 1983, the group in La Côte D'Ivoire had reported their results of the first 300 cases of open heart surgery performed in Abidjan [4]. By 1987, this group published data on 851 open heart operations performed at the same institution in Abidjan [5]. In Senegal, four patients underwent open heart surgery in 1995 at the Aristide Le Dantec hospital in Dakar. Currently, open-heart surgery is performed regularly in Senegal, with their center serving as a reference point for neighboring countries.

Our experience with open-heart surgery in Ghana began in 1964 when Charles Odamtten Easmon, a Ghanaian surgeon used surface cooling as a means of achieving hypothermia to close atrial septal defects in two patients. Subsequently, a myriad of problems stalled the open-heart program in Ghana until 1989 when Ghana's National Cardiothoracic Center (NCTC) was set up by Dr. Kwabena Frimpong-Boateng who was a pioneer of heart transplantation at the Medizinische Hochschule Hannover in Germany where he trained. The NCTC currently performs over 400 cardiothoracic operations a year, roughly a quarter of these are open-heart procedures performed using extracorporeal circulation. Operations performed include surgery for congenital heart disease, rheumatic heart disease, coronary artery bypass graft, and many others. In our 22 years of existence, the NCTC has received referrals from several West African nations including Cameroon and has reported results of open-heart procedures performed on patients from across the West African sub-region [6,7]. We are equipped with two ultra-modern theatres, intensive care unit, high-dependency unit, wards, an executive suite facility for VIP admissions, cardiac catheterization, echocardiography service, a dedicated laboratory for biochemical and hematological tests, radiography and a dedicated renal dialysis unit. Our facilities equip us to offer almost the whole spectrum of cardiac and thoracic operations and we have served as a tertiary referral center for cardiothoracic pathology for much of West Africa. We started a video-assisted thoracoscopic surgery (VATS) service in 2008. Accordingly, the NCTC has been accredited by the West African College of Surgeons as a center of excellence for the training of cardiothoracic surgeons for West Africa. We have trained a total of five Ghanaian cardiothoracic surgeons with a sixth near completion of training. In addition, more than 20 cardiothoracic surgeons from across West Africa have received training at the NCTC [8]. We have made significant contributions to the international literature and have pioneered several innovative surgical operations [9,10] in both cardiac and thoracic surgery which space does not allow for enumeration.

It is surprising to us therefore when Budzee and colleagues claim that their institution is the only cardio-surgical center in Central / West Africa, equipped with ultra modern health technologies to offer diverse services. We have been in this service since 1989 and have made and continue to make significant contributions in the sub-region and beyond. We know other centers in the sub-region that have also done quite well until recently.

We congratulate Budzee and coworkers for the effort they have made to bring open-heart surgery to Cameroonians. We are well aware of the many obstacles and challenges facing developing countries starting open-heart surgery programs but we consider their report overzealous and historically inaccurate. We wish to emphasize that theirs is not the only cardio-surgical center in West Africa; and coronary artery bypass grafts have been performed (and still being performed) by several indigenous West African teams starting two to three decades before their own.

The authors declared no conflicts of interest.
